# Intrauterine Growth Restriction Causes Abnormal Embryonic Dentate Gyrus Neurogenesis in Mouse Offspring That Leads to Adult Learning and Memory Deficits

**DOI:** 10.1523/ENEURO.0062-21.2021

**Published:** 2021-10-08

**Authors:** Ashley S. Brown, Matthew Wieben, Shelby Murdock, Jill Chang, Maria L. V. Dizon, Mark St. Pierre, Raul Chavez-Valdez, Richard I. Dorsky, Camille M. Fung

**Affiliations:** 1 Division of Neonatology, Department of Pediatrics, University of Utah School of Medicine, Salt Lake City, Utah 84158; 2 University of Utah, Salt Lake City, Utah 84108; 3 Division of Neonatology, Department of Pediatrics, Northwestern University, Chicago, Illinois 60611; 4 Division of Neonatology, Department of Pediatrics, Johns Hopkins University, Baltimore, Maryland 21287; 5 Department of Neurobiology and Anatomy, University of Utah, Salt Lake City, Utah 84108

**Keywords:** embryonic dentate gyrus neurogenesis, fetal growth restriction, hypertensive disease of pregnancy, intrauterine growth restriction, learning and memory, neural stem cell

## Abstract

Human infants who suffer from intrauterine growth restriction (IUGR), which is a failure to attain their genetically predetermined weight, are at increased risk for postnatal learning and memory deficits. Hippocampal dentate gyrus (DG) granule neurons play an important role in memory formation; however, it is unknown whether IUGR affects embryonic DG neurogenesis, which could provide a potential mechanism underlying abnormal postnatal learning and memory function. Using a mouse model of the most common cause of IUGR, induced by hypertensive disease of pregnancy, we first assessed adult learning and memory function. We quantified the percentages of embryonic hippocampal DG neural stem cells (NSCs) and progenitor cells and developing glutamatergic granule neurons, as well as hippocampal volumes and neuron cell count and morphology 18 and 40 d after delivery. We characterized the differential embryonic hippocampal transcriptomic pathways between appropriately grown and IUGR mouse offspring. We found that IUGR offspring of both sexes had short-term adult learning and memory deficits. Prenatally, we found that IUGR caused accelerated embryonic DG neurogenesis and Sox2^+^ neural stem cell depletion. IUGR mice were marked by decreased hippocampal volumes and decreased doublecortin^+^ neuronal progenitors with increased mean dendritic lengths at postnatal day 18. Consistent with its known molecular role in embryonic DG neurogenesis, we also found evidence for decreased Wnt pathway activity during IUGR. In conclusion, we have discovered that postnatal memory deficits are associated with accelerated NSC differentiation and maturation into glutamatergic granule neurons following IUGR, a phenotype that could be explained by decreased embryonic Wnt signaling.

## Significance Statement

Postnatal learning and memory deficits are common in infants born with intrauterine growth restriction (IUGR). However, the embryonic cellular and molecular hippocampal changes are elusive compared with the many postnatal studies in the literature. Using a translationally relevant mouse model with short-term memory deficits, we discovered that IUGR accelerated embryonic hippocampal dentate gyrus (DG) neurogenesis along with NSC depletion. Decreased postnatal hippocampal volumes with altered neuronal progenitor development were noted. Transcriptomic analysis revealed decreased Wnt signaling as a strong candidate for embryonic cellular changes after IUGR. To our knowledge, this is the first investigation linking IUGR-induced embryonic hippocampal DG alterations with postnatal neuronal developmental aberrations. This model provides a framework to investigate other hippocampal domains and cell types that modulate neuronal maturation and memory function.

## Introduction

Intrauterine growth restriction (IUGR) describes a condition of fetal weight below the genetically predetermined potential (Resnik, 2002) and is usually detected clinically when fetal growth parameters are incongruent with gestational age-matched normative values. In high-income countries, IUGR complicates 3–9% of all pregnancies, whereas the incidence is sixfold higher in low-income countries (Lee et al., 2017). The etiologies of IUGR are numerous, relating to maternal, placental, and cord abnormalities, or fetal factors. The primary cause in developed countries is hypertensive disease of pregnancy (HDP), which creates uteroplacental insufficiency whereby the placenta fails to support fetal growth. IUGR infants are prone to a range of health problems that affect virtually every organ including the brain, culminating in impairment in motor, sensory, cognitive, and learning and memory tasks (Leitner et al., 2007).

The hippocampus, a medial cortical structure required for forming new memories and learned behaviors (Eichenbaum, 2000), is divided into the dentate gyrus (DG) and cornu ammonis (CA) fields in both humans and rodents, and is particularly susceptible to IUGR injury (Gilchrist et al., 2018). Both children and animal models with IUGR have shown reductions in hippocampal volume by magnetic resonance imaging and correlated poorer memory performance (Isaacs et al., 2000; Mallard et al., 2000; Lodygensky et al., 2008; Camprubí Camprubí et al., 2017). Multiple experimental models of IUGR have also indicated that both the neuron number and dendritic–axonal morphologies within the DG, CA1, and CA3 are altered following uteroplacental insufficiency. For example, guinea pig neonates with IUGR have fewer cells in the CA1 region (Mallard et al., 2000), consistent with reduced cell numbers in CA1 pyramidal layer in juvenile rats with IUGR (Camprubí Camprubí et al., 2017). Furthermore, a reduction in dendritic length and outgrowth, reduced branch numbers of apical dendrites, and alterations in basal dendritic branch points in the CA1 region have been reported (Dieni and Rees, 2003). What is unknown from existing literature are the *in utero* changes that lead to the functional and cellular differences observed in postnatal life.

We therefore used the mouse model of IUGR of our laboratory, which was induced by thromboxane A_2_-analog infusion to produce HDP (Fung et al., 2011; Gibbins et al., 2018), to dissect at the cellular and molecular events that are disrupted during embryonic DG neurogenesis. We focused on the DG because information from the entorhinal cortex to be encoded into memory enters here first before further processing in the CA regions (Amaral et al., 2007). We noted sex differences throughout the study. We hypothesized that IUGR would alter DG glutamatergic neuronal development beginning at neurogenesis through migration to the developing DG anlage, resulting in abnormal proportions of maturing glutamatergic neurons at birth. After birth, ongoing postnatal DG neuronal maldevelopment would result in hippocampal volume loss, and in learning and memory deficits in adult life.

To test our hypothesis, we first interrogated whether our IUGR mouse offspring showed adult learning and memory deficits. We then explored the effects on embryonic DG neurogenesis by systematically quantifying the percentages of Sox2^+^ neural stem cells (NSCs) that give rise Tbr2^+^ intermediate neuronal progenitors (INPs), NeuroD^+^ NPs, and Prox1^+^ immature and mature glutamatergic granule neurons (Seki et al., 2014) during early and late IUGR. After birth, we quantified hippocampal volumes at postnatal day 18 (P18) and P40 correlating with middle and completed ages of hippocampal DG development. We additionally quantified doublecortin^+^ (DCX^+^) neuronal committed progenitor cells/neuroblasts and Calbindin 1^+^ (Calb1^+^) neurons in the DG at P18 and P40. Finally, we performed RNA sequencing (RNA-seq) in appropriately grown and IUGR embryonic hippocampi to determine the differential transcript expression and decipher candidate pathways that may be associated with the cellular phenotypes.

We found that adult IUGR offspring of both sexes displayed memory deficits during short-term recall, with IUGR females showing more compromise over IUGR males. This is seen in the setting of both sexes having premature embryonic DG neurogenesis and NSC depletion during IUGR. Postnatally, we documented decreased hippocampal volume and noted IUGR females to have the lowest volumes. We additionally showed that DG DCX^+^ neuronal committed progenitor cells/neuroblasts had smaller cell volumes but higher mean dendritic lengths at P18 that normalized in morphology at P40 in both sexes. We saw no differences in DG Calb1^+^ neurons. We also discovered that decreased Wnt signaling during IUGR as a logical molecular candidate to account for premature DG neurogenesis and NSC depletion. To our knowledge, this is the first description of IUGR-induced embryonic cellular and molecular changes in the hippocampal DG. These changes result in altered postnatal neuronal development, leading to learning and memory deficits in adulthood.

## Materials and Methods

### Experimental designs

#### Mouse model of IUGR

All animal procedures were approved by the University of Utah Animal Care Committee. Full details can be found on model inception (Fung et al., 2011). Briefly, we set up timed matings of wild-type C57BL/6J mice (catalog #000664, The Jackson Laboratory). The morning after noting a vaginal plug denoted embryonic day 0.5 (E0.5). At E12.5 (term gestation, ∼20 d), pregnant dams were anesthetized and micro-osmotic pumps (0.5 μl/h; catalog #1007D, DURECT) containing either vehicle (0.5% ethanol, a sham control, which generates appropriately grown offspring) or 4000 ng/μl U-46 619 (a thromboxane A_2_-analog, an experimental group, which generates IUGR offspring) dissolved in vehicle (catalog #16 450, Cayman Chemical) were implanted retroperitoneally. We have previously shown that the brain white matter characteristics in the vehicle group exposed to 0.5% ethanol were similar to those in pups exposed to normal saline as a vehicle (Chang et al., 2018). We chose to use 0.5% ethanol as the solubility of U-46619 is superior in 0.5% ethanol than in saline according to manufacturer recommendation. Pups used for embryonic studies were delivered via cesarean sections after maternal anesthesia at E15.5 or E19. Pups used for postnatal studies were born vaginally. All sham control and IUGR pups were cross-fostered to unmanipulated dams to minimize surgery-related complications. We previously established that mouse dams who received U-46619 developed maternal hypertension within 24 h of pump implantation, with mean blood pressures 20% higher than those in sham-operated dams (Fung et al., 2011). Additionally, IUGR offspring exhibited smaller weight gain from E17.5 to E19 and were 15% symmetrically growth restricted at birth compared with sham controls (Fung et al., 2011; Gibbins et al., 2018). Following the weight curves over the first year of life, we have previously established that IUGR males and females weighed less than their age- and sex-matched shams from P1 to P21. However, at P28, IUGR males caught up in weights with sham males, whereas IUGR females caught up in weights to sham females at P77. From P238 to the end of the first year, IUGR males weighed significantly more than age-matched sham males versus IUGR females weighing similarly to sham females (Fung et al., 2011). This pattern of weight gain was duplicated in our current cohort of mice used for experimentation.

#### Behavioral assays

##### Novel object recognition

We habituated 3-month-old sham and IUGR mice of both sexes (*n* = 10/each treatment and sex) to the test arena (a black box measuring 40 × 40 cm equipped with a camera overhead that allowed for the filming of mouse exploration) for 15 min on day 1. The next day, we placed two identical objects in opposite corners of the arena and mice were allowed to explore for 15 min. The duration of time spent exploring either object minus the time spent climbing on the object (which does not constitute exploration) was recorded with a camera and calculated using EthoVision 3.1 (Noldus). On the third day, we substituted one of the old objects with a novel object, and mice were allowed to explore for 15 min. The discrimination index was calculated from the duration of time spent with the novel object subtracting from the time spent with the old object divided by the total time spent with novel and old objects (Rodriguiz and Wetsel, 2006).

##### Fear conditioning

Six-month-old sham and IUGR mice of both sexes (*n* = 6/each treatment and sex) were placed into Plexiglas conditioning chambers on day 1 for five trials of a 30 s tone at 75 dB with a 20 s trace interval, followed by a brief 1 s mild footshock at 0.7 mA. Mice were returned to the same chamber the next day for 5 min and scored for time spent in freezing motion in the absence of tone in contextual conditioning. They were then placed into a novel chamber for 5 min and scored for time spent in freezing motion with the presentation of the tone but without a footshock in cued test (Rodriguiz and Wetsel, 2006).

#### Brain perfusion and immunohistochemistry

Pregnant mouse dams at E15.5 or E19 or postnatal mice were sedated with ketamine (80–100 μg/g) and xylazine (7.5–16 μg/g). Once adequate anesthesia was achieved by lack of withdrawal on toe pinch, a thoracotomy was performed to expose the heart. A 25 ga needle was inserted into the left ventricle, and the animal was perfused with normal saline followed by perfusion with 4% paraformaldehyde (PFA). A cesarean section was performed to deliver pups for embryonic time points. Pup brains were extracted and postfixed in additional 4% PFA for 1 h at room temperature (RT). After washing with PBS, offspring brains were cryoprotected with increasing concentrations of sucrose at 10%, 20%, and 30% each time overnight and embedded in OCT (optimal cutting temperature) medium. Brains were stored at −80°C until cryosectioning at 12 μm. All coronal brain sections from the beginning of the dorsal to ventral hippocampus, defined as when the fasciola cinereum appeared in proximity to the dorsal third ventricle and the end of the dorsal subiculum and before the CA3 descended inferiorly to the horizontal level of the fasciculus retroflexus, were collected in a series of 20 slides such that the 1st, 21st, and 41st sections were on the first slide, while the 2nd, 22nd, and 42nd sections were on the second slide, etc.

For immunofluorescent (IF) staining of single cell types in embryonic brains, we hydrated each slide spanning the dorsal hippocampal DG with 1× PBS and proceeded with antigen retrieval with neutral buffer at 37°C × 30 min for Sox2, 10 mm citrate buffer, pH 6.0, at 37°C × 30 min for Tbr2, neutral buffer at 85°C × 10 min for NeuroD, and 10 mm citrate buffer, pH 6.0, at 95°C × 5 min for Prox1 (neutral buffer; catalog #CTS016, R&D Systems). Sections were then blocked with 10% or 5% (for Tbr2) normal goat serum (NGS) at RT for 1 h. We incubated with the following primary antibodies at 4°C overnight: Sox2 (1:300; catalog #AB5603, Millipore Sigma), Tbr2 (1:500; catalog #ab23345, Abcam), NeuroD (1:100; catalog #4373S, Cell Signaling Technology), and Prox1 (1:1000; catalog #AB5475, Millipore Sigma). After washing, we incubated with 1:1000–1:2000 goat anti-rabbit secondary antibody, Alexa Fluor 594 (catalog #A11037, Thermo Fisher Scientific), at RT for 1 h. We applied nuclear 4′,6′-diamidino-2-phenylindole dihydrochloride (DAPI) counterstain and added Fluoromount-G (catalog #0100–01, SouthernBiotech) before all slides were coverslipped for imaging (*n* = 6–7/each treatment and sex).

##### 5-Ethynyl-2′deoxyuridine labeling of embryonic brains

5-Ethynyl-2′deoxyuridine (EdU) is a nucleoside analog of thymidine and is incorporated into DNA during active DNA synthesis (S phase of the cell cycle). Detection is based on a click reaction, a copper-catalyzed covalent reaction between an azide, which is the Alexa Fluor 488 dye, and an alkyne, which is the EdU (catalog #C10339, Thermo Fisher Scientific). EdU is capable of crossing the placenta to be taken up by actively dividing neural stem and progenitor cells (NSPCs) in the fetal brain. We injected 100 μg/g EdU for 2 h before pregnant dam harvest. Pup brains were processed as noted above. We followed the manufacturer protocol for EdU detection.

##### Double-labeling IF-immunohistochemistry for EdU and phosphohistone H3 at serine 10 of embryonic brains

We hydrated brain sections of interest with 1× PBS and followed the EdU labeling protocol using Alexa Fluor 594 as described previously. Once the EdU labeling protocol was completed, we blocked the sections with 5% NGS at RT for 1 h protected from light exposure. We then incubated with rabbit phosphohistone H3 (pHH3) serine 10 (Ser10) antibody at 4°C overnight at 1:200 (catalog #9701, Cell Signaling Technology) in dark. After washing, we incubated with 1:1000 goat anti-rabbit secondary antibody, Alexa Fluor 488 (catalog #A32723, Thermo Fisher Scientific) at RT for 1 h. After further washing, we applied nuclear DAPI counterstain and added Fluoromount-G (catalog #0100-01, SouthernBiotech) before all slides were coverslipped for imaging.

##### Double-labeling IF-immunohistochemistry for DCX and Calb1 in postnatal brains

After cryoprotection in 30% sucrose, perfused brains were flash frozen using 2-methylbutane and stored at −80°C. A freezing microtome was used to cut brains coronally into 50 μm sections. For these experiments, coronal brain sections were classified as anterior, containing dorsal hippocampus, again if the fasciola cinereum appeared in proximity to the dorsal third ventricle and the end of the dorsal subiculum and before the CA3 descended inferiorly to the horizontal level of the fasciculus retroflexus. Anterior coronal sections were washed in TBS, pH 7.2, for 10 min followed by antigen retrieval with sodium citrate buffer, pH 6.0, for 90 min at 80°C. Tissues were permeabilized using 0.4% Triton X in TBS for 15 min at RT, followed by blocking using 10% NGS in 0.1% Tween/TBS for 1 h at RT, before exposure of primary antibodies in the following combination: (1) chicken anti- DCX IgY (2.5 μg/ml; catalog #ab153668, Abcam); and(2) rabbit monoclonal IgG anti-Calb1 IgG (2.5 μg/ml; catalog #D1I4Q, Cell Signaling Technology). Primary antibodies were mixed in 4% NGS in TBS-T for overnight incubation at 4°C, one at the time. Following overnight incubation with the last primary antibody, sections were washed in TBS and exposed in the dark for 2 h at RT to goat anti-chicken IgY Alexa Fluor 488 and goat anti-rabbit IgG Alexa Fluor 568, emitting green and red fluorescence signal, respectively (Thermo Fisher Scientific), mixed in 4% NGS/TBS-T. Last, tissues were incubated for 5 min in DAPI (1 μg/ml) in TBS, washed in TBS, and mounted and dried for 30 min before being coverslipped with ProLong Glass Antifade Mountant (Thermo Fisher Scientific).

All antibodies used for immunohistochemistry experiments were tested for specificity. Negative controls included no primary antibody and subtype- and species-specific Ig replacing primary antibody at identical concentrations.

##### Imaging and quantification of cells

All sections were imaged using a laser-scanning confocal microscope (Olympus) with a 20× objective lens and Fluoview FV1000 software (Olympus). We chose 20× magnification to capture the entire developing DG for cell quantification. The individual performing the quantification was blinded to the treatment groups. Once images were obtained, we used NIH ImageJ software to split the multichannel images into blue (DAPI), green, or red channels individually and to manually outline the hippocampal DG area at E15.5 beginning at the ventricular zone (VZ) down to the dentate notch and across to the pial surface of the hippocampal formation. For E19, we manually outlined the dentate anlage from the ventricular zone to developing C-shaped dentate anlage on the pial side. Images were thresholded to obtain the optimal exposure for each image such that each nucleus is highlighted in red. The number of blue DAPI^+^ nuclei representing the total number of cells was quantified within the area using the Analyze Particles macro. The number of red Sox2^+^, Tbr2^+^, NeuroD^+^, Prox1^+^, or green EdU^+^ cells were counted similarly using the Analyze Particles macro and were expressed as a percentage of total DAPI^+^ cells. Data are expressed as percentages of Sox2^+^, Tbr2^+^, NeuroD^+^, Prox1^+^, or EdU^+^ cells within the DG.

For quantification of double-labeled EdU^+^ and pHH3^+^ cells, we imaged all sections with a 20× objective lens and outlined the area of the developing DG as previously described in ImageJ. We maximized the brightness of all red EdU^+^ cells and green pHH3^+^ cells in their individual channels in TIFF images to negate background fluorescence. We merged the red and green channels to create an image of colocalization. We then converted the image into RGB color and used Color Threshold to include all cells with yellow color as colocalization. We thresholded yellow color into black and white, and, given the sparse colabeling, we were able to count individual cells manually.

For quantification of double-labeled DCX^+^ and Calb1^+^ cells, four-tiled stitched *z*-stacks of 0.9 μm sections were taken at 1024 × 1024 pixels, 16 bit depth, and averaged 2×, captured using a Plan-Apochromat 63×/1.4 oil differential interference contrast M27 objective and 0.7 zoom to produce an uncompressed image of 290.33 × 290.33 μm presenting between ∼25% and ∼40% of the entire dorsal DG contained in the coronal section from the most external edge of the granular cell layer of the apex. The *z*-stacks were set for slicing at 1 air unit (0.9 μm) to the maximal wavelength used in the experiments (639 nm) using a laser-scanning confocal microscope (model LSM 700 AxioObserver, Carl Zeiss). The same pinhole, gain, and offset setting were applied to all images using the identical imaging configurations. The detection wavelengths used included 300–483 nm for DAPI, 493–550 nm for Alexa Fluor 488 (DCX), and 560–600 nm for Alexa Fluor 568 (Calb1). The *z*-stacks were saved in .czi format for uncompressed processing. Bitplane AG Imaris x64 version 9.7.2 software was used for image processing. Visualization control of objects was gained in surpass mode. The nuclear DAPI stain has been made non-nuclear under the Imaris “DAPI surface reconstruction” mode, which created a bubble-like object where the three-dimensional limits of the nuclei were rendered for quantification. An automatic creation algorithm was used for processing and volumetric analysis. Following the definition of channel sourcing and the level of surface detailing, the surface renderings were detected and adjusted automatically with background subtraction, and masking to zero voxels inside the surface were applied. The split touching objects function was used with the seed point diameter set to the average diameter of the cells of interest (DCX^+^ or Calb1^+^). Average values statistics for cell count, volume, and intensity sum for analysis were retrieved. For dendritic complexity analysis for DCX-expressing cells, an automatic dendrite detection protocol was used. The green channel was selected in the display adjustment window, followed by the creation of new filaments starting with the Autopath algorithm setting to calculate the diameter of filament from the image. The region of interest limited by most outer layer of the granular cells and excluding the hilus of the DG. Diameter of the starting points (the largest dendritic diameter) and seed points (the smallest dendrite) were determined using slice mode. These points were then detected and adjusted using thresholds automatically. The option to remove seed points around a set diameter of the starting point sphere region to avoid excess or false filament creation around high-intensity regions, such as the soma, was used. Detected points were then automatically connected with lines following the image intensity. Detailed average value statistics were then exported for analysis.

#### RNA sequencing

We dissected E15.5 sham and IUGR hippocampi of both sexes (*n* = 4/each treatment) from whole mouse brains. Total RNA was extracted, followed by verification of RNA quality and quantity by Agilent ScreenTape Assay at the University of Utah High-Throughput Genomics core facility. The RNA sequencing library was made using the Illumina TruSeq Stranded Total RNA Sample Prep Kit with Ribo-Zero Gold, which allowed for the removal of cytoplasmic and mitochondrial rRNA. Remaining RNA was chemically fragmented and random primed for reverse transcription to construct cDNA libraries. The average insert size of libraries was ∼150 bp, with inserts ranging from 100 to 400 bp. Once the libraries were made and validated, sequencing adapters were added and sequenced as single reads using the HiSeq 2500 platform.

Reads were aligned to *Mus musculus* genome assembly GRCm38 (mm10, Genome Reference Consortium). Of the 16,873 genes contained in the mouse genome, we determined the differential expression of protein-coding transcripts that had ≥10 but <50,000 counts between sham control and IUGR hippocampi. Using a false discovery rate of 13 (*p* ≤ 0.05) and log_2_ ratios greater than or equal to +1 or less than or equal to −1 (twofold increase or decrease in gene expression), 611 protein-coding transcripts were found to be differentially expressed. We entered these differentially expressed genes into ingenuity pathway analysis (IPA; Qiagen Bioinformatics) to determine canonical pathways that were aberrantly expressed in IUGR.

**Figure 8.**
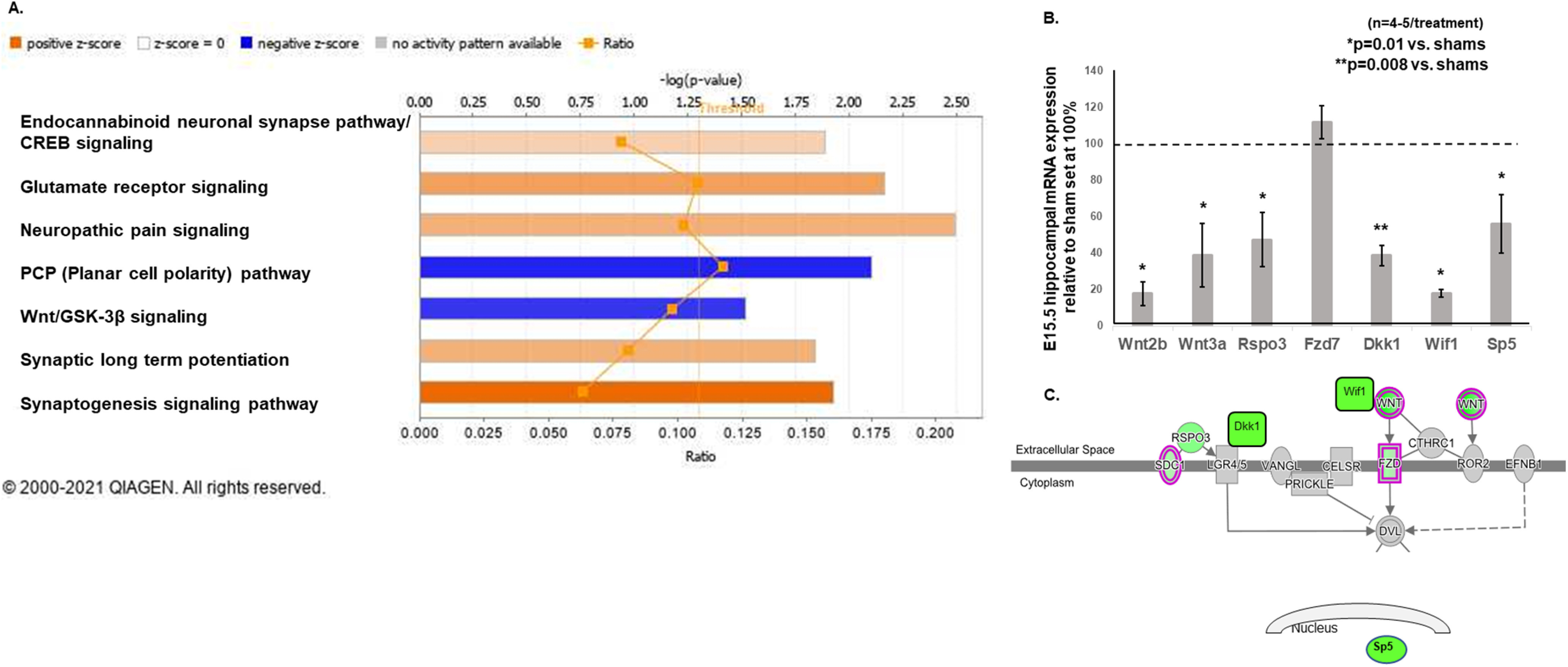
***A***, Ingenuity Pathway Analysis of the 611 differentially expressed protein-coding genes to determine canonical pathways that were altered in IUGR at E15.5. We used *z* scores >1.0 as the cutoff to determine predicted downregulated or upregulated activity. The canonical Wnt/GSK-3β and noncanonical (PCP) pathways were predicted to have downregulated activity in IUGR (blue bars denoting negative *z* scores determined by the IPA Canonical Pathway algorithm, which infers likely deactivation states of biological functions based on comparison with a model that assigns random regulation directions). The glutamate receptor signaling pathway, which shared the same differentially expressed genes as the endocannabinoid neuronal synapse pathway, CREB signaling, neuropathic pain signaling pathway, synaptic long-term potentiation, and synaptogenesis signaling pathway, was predicted to have upregulated activity in IUGR (orange bars denoting positive *z* scores determined by the IPA Canonical Pathway algorithm, which infers likely activation states of biological functions based on comparison with a model that assigns random regulation directions). Pathways with –log(*p* value) above the orange threshold line denoted a significance of *p* ≤ 0.05. Ratio signified the number of genes detected in RNA-seq in that pathway divided by the number of genes known in that pathway. ***B***, Genes of canonical and noncanonical Wnt (PCP) signaling pathways discovered from RNA-seq were validated with qRT-PCR in a separate cohort of E15.5 hippocampi. IUGR reduced hippocampal gene transcripts of *Wnt2b* and *Wnt3a* ligands, *Rspo3* ligand for the PCP pathway, *Dkk1* and *Wif1* inhibitors, and *Sp5*, a readout of the canonical pathway. Sham values were normalized to 100%, and IUGR-induced changes were expressed as percentages of sham values. *p = 0.01 compared with sham controls. **p = 0.008 compared with sham controls. Student’s *t* test was used to compare sham and IUGR offspring. No differences were noted when sex was separated. ***C***, A representative diagram showing the localization of canonical and noncanonical (PCP) signaling gene transcripts that were decreased by IUGR. Green color within each gene denoted decreased gene expression in the analysis.

We picked the Core Analysis for our dataset. General settings were set to have our reference set match the Ingenuity Knowledge Base (genes only) showing direct and indirect relationships. We additionally chose Networks Interaction and Causal to include 35 molecules/network plus 25 networks/analysis. We specified “mouse” under “Species,” “nervous system” under “Tissues and Cell lines,” “Log ratios” of “greater than or equal to +1 or less than or equal to −1,” and “Intensity/RPKM/FPKM” of “13.” Pathways with –log(*p* value) above the threshold (see Fig. 8, right of the vertical orange line) denoted the significance of *p* ≤ 0.05 based on the IPA Canonical Pathway algorithm, which determined that pathways overlap based on molecules in common. Positive or negative *z* scores are determined based on likely activation or deactivation states of biological functions compared with a model that assigns random regulation directions. We used a *z* score of 1.0 to include pathways that predicted a positive or negative directionality of activity. The ratio in each pathway signified the number of genes detected in RNA-seq divided by the number of genes known in that pathway (see Fig. 8). RNA sequencing data have been deposited in the NCBI Gene Expression Omnibus (GEO) and are accessible through GEO Series accession number GSE173845 (https://www.ncbi.nlm.nih.gov/geo/query/acc.cgi?acc=GSE173845).

#### Quantitative real-time PCR

Genomic-free total RNA was isolated from a separate cohort of E15.5 sham and IUGR hippocampi (*n* = 4–5/each treatment) using Nucleospin RNA protocol (Takara Bio USA). Total RNA was quantified with a spectrophotometer (catalog #ND-1000, Nanodrop). cDNA was synthesized from 4 μg of total RNA using random hexamers of SuperScript III First-Strand Synthesis System (Thermo Fisher Scientific). Using real-time quantitative reverse transcription PCR (qRT-PCR) as previously described (Fung et al., 2011), we examined mRNA levels of Wnt signaling genes (*Wnt2b, Wnt3a, Rspo3, Fzd7, Dkk1, Wif1, Sp5*) to corroborate with RNA-seq data. All primers and probes were purchased as TaqMan Assays-on-Demand with assay identification numbers Mm00437330_m1 (*Wnt2b*), Mm00437337_m1 (*Wnt3a*), Mm01188251_m1 (*Rspo3*), Mm00433409_s1 (*Fzd7*), Mm00438422_m1 (*Dkk1*), Mm00442355_m1 (*Wif1*), and Mm00491634_m1 (*Sp5*; Thermo Fisher Scientific). Each sample was run in quadruplicate. Relative quantitation comparing 2^-ΔCt^ values was used to analyze changes in mRNA expression between sham control and IUGR using *GAPDH* as a control. We have previously validated that *GAPDH* was an appropriate control using parallel serial dilutions between sham control and IUGR cDNA and that the amplification efficiencies between the target genes and *GAPDH* were comparable.

### Statistical analyses

Data are expressed as the mean ± SEM for continuous variables or sum ranks ± mean ranks for noncontinuous variables. Normality of data with Kolmogorov–Smirnov test and equality of variances with *F* test were performed to determine whether parametric tests were appropriate. Student’s *t* test was used to analyze mean effects for two groups. Two-way ANOVA with Tukey’s HSD *post hoc* test was used to examine the effects of treatment (sham control or IUGR), sex, or both sex and treatment on main effects. Mann–Whitney *U* test was used for nonparametric testing. Statistical significance was set at *p* < 0.05 using STATVIEW software (SAS Institute).

## Results

### IUGR impairs adult hippocampal recognition memory

Recognition memory was tested to determine the effects of IUGR on adult mouse hippocampal function. Memory can be classified as either implicit or explicit. Implicit memory refers to nonconscious learning that is evident through performance and does not require access to any conscious memory contents (Schacter et al., 1998). Explicit memory, on the other hand, involves encoding, storage, and retrieval that is recalled with conscious effort and is highly plastic, permitting the creation of new associations (Kandel and Iversen, 2000). Object interaction/recognition is an implicit memory test, whereas fear conditioning is an explicit memory test.

**Figure 1.**
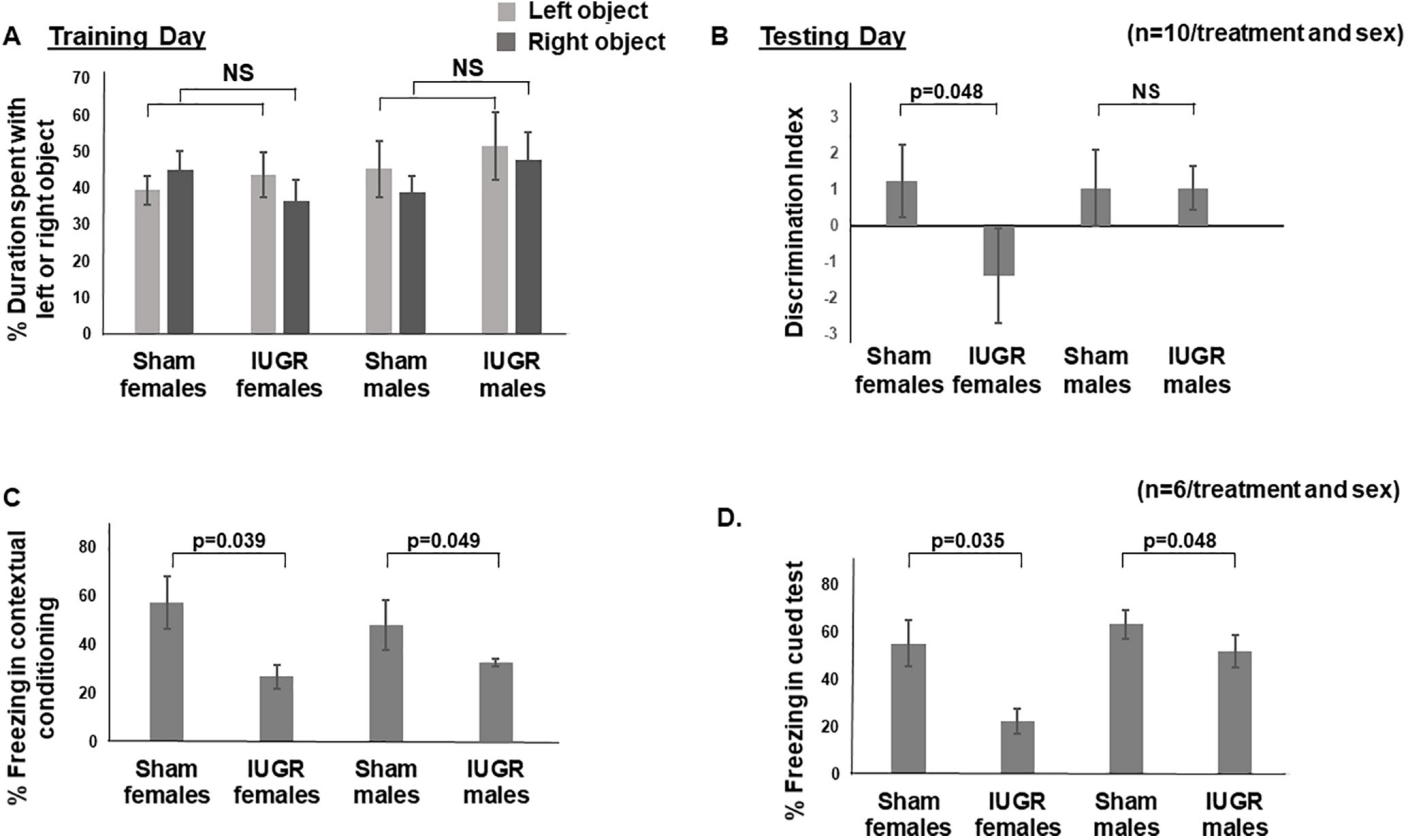
***A***, Percentage of duration of time spent with the left or right object on training day. Light gray bars denote the left object. Dark gray bars denote the right object. Sham and IUGR offspring spent an equal duration of time exploring left and right objects without sex differences. NS, No significance. ***B***, Discrimination index [(time spent with novel object – time spent with old object)/time spent with novel object plus time spent with old object)] on testing day. +1, More time spent with the novel object; 0, no preference; −1, More time spent with the old object. IUGR females spent more time with the old object, demonstrating a lack of memory to the exposure of the old object from previous day compared with sham females. *p* = 0.048, IUGR females versus sham females. ***C***, Percentage of time spent freezing in contextual conditioning. IUGR offspring of both sexes spent less time freezing when the audible tone was heard but no footshock followed. *p* = 0.039, IUGR females compared with sex-matched sham controls; *p* = 0.049, IUGR males compared with sex-matched sham controls. ***D***, Percentage of time spent freezing in cued test. IUGR offspring of both sexes spent less time freezing when placed in the same chamber in which a previous audible tone gave rise to a footshock. *p* = 0.035, IUGR females compared with sex-matched sham controls; *p* = 0.048, IUGR males compared with sex-matched sham controls. Two-way ANOVA with Tukey’s HSD *post hoc* test was used to examine the effects of treatment (sham or IUGR), sex, or both sex and treatment on each main effect (percentage of time spent exploring left or right object, discrimination index, percentage of time freezing in contextual conditioning or cued test).

We noted no difference in the duration of time spent with the left or right object during training day (Fig. 1*A*). When we substituted one old object with a novel object on the next day, IUGR females spent more time exploring the old object rather than the novel object compared with sham females (*p* = 0.048; Fig. 1*B*). IUGR males, on the other hand, explored the novel object as frequently as sham males (Fig. 1*B*). When we assayed fear conditioning, both IUGR females and males showed less freezing time or fear during both contextual conditioning (females, *p* = 0.039; males, *p* = 0.049; Fig. 1*C*) and cued tests (females, *p* = 0.035; males, *p* = 0.048; Fig. 1*D*) when compared with sex-matched sham controls.

### IUGR reduces embryonic hippocampal neural stem cells and induces excessive neuronal differentiation

**Figure 2.**
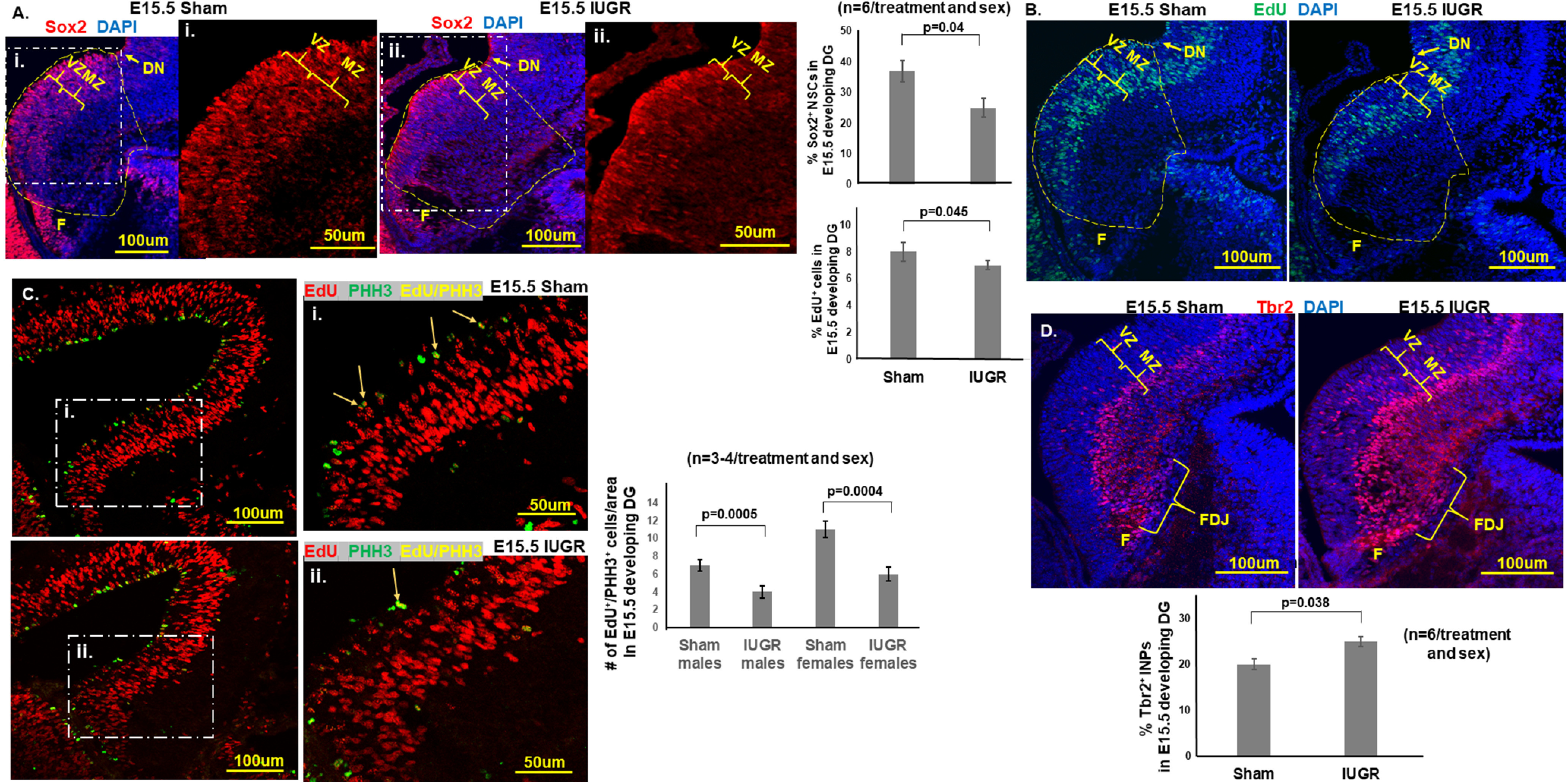
***A***, Representative photomicrographs of Sox2^+^ NSCs in E15.5 developing DG. Blue DAPI stain denoted cell nuclei. i, ii, Magnified views of Sox2^+^ NSCs in the white dashed regions of ***A***. IUGR offspring of both sexes showed decreased percentages of Sox2^+^ NSCs (number of Sox2^+^ cells divided by the number of DAPI^+^ cells in the yellow dashed outlined area) in the VZ and MZ. *p* = 0.04, IUGR males and females compared with sex-matched shams. F, Fimbria. Yellow dashed outlines delineate the quantified areas that were manually traced in ImageJ beginning at the dentate notch (DN) to the beginning of the fimbria on the ventricular side and across to the pial surface. ***B***, Representative photomicrographs of EdU^+^ proliferative cells in E15.5 developing DG. Blue DAPI stain denoted cell nuclei. IUGR offspring of both sexes showed decreased percentages of EdU^+^ proliferative cells (the number of EdU^+^ cells divided by the number of DAPI^+^ cells in the yellow dashed outlined area; i.e., cells in S phase of cell cycle, in VZ and MZ. *p* = 0.045, IUGR males and females compared with sex-matched sham controls. Student’s *t* test was used to compare sham and IUGR offspring for Sox2 and EdU, given that no differences were noted when sex was separated. ***C***, Representative photomicrographs of E15.5 sham and IUGR developing DG with EdU and pHH3 at Ser 10 immunofluorescent costaining. White dashed boxed areas denoted as i and ii are enlarged regions demonstrating the colocalization of red EdU^+^ cells with green pHH3^+^ cells in yellow (arrows). Both IUGR males (*p* = 0005) and females (*p* = 0.0004) had a decreased number of EdU^+^/PHH3^+^ colocalized cells compared with sex-matched shams showing that IUGR progenitors had fewer cells completing mitosis of the cell cycle. Two-way ANOVA with Tukey’s HSD *post hoc* test was used to examine the effects of treatment (sham or IUGR), sex, or both sex and treatment on the number of EdU^+^/pHH3^+^ colocalized cells. ***D***, Representative photomicrographs of Tbr2^+^ in E15.5 developing DG. Blue DAPI stain denoted cell nuclei. IUGR offspring of both sexes showed increased percentages of Tbr2^+^ INPs (the number of Tbr2^+^ cells divided by the number of DAPI^+^ cells) in the VZ, MZ, and FDJ. *p* = 0.038, IUGR males and females compared with sex-matched sham controls. Student’s *t* test was used to compare sham and IUGR offspring given no differences were noted when sex was separated.

The caudomedial cortical primordium possesses intrinsic cues to generate hippocampal neurons at E10.5 in mice. By E12.5, hippocampal field patterning appears to be well established (Tole and Grove, 2001). In our model of HDP, which is induced at E12.5, we noted that by E15.5, IUGR offspring of both sexes had a decreased percentage of Sox2^+^ NSCs among the total number of DAPI^+^ cells in the VZ compared with sham controls (*p* = 0.04; Fig. 2*A*). The total absolute number of DAPI^+^ cells were similar between E15.5 sham and IUGR DG (sham males, 1842 ± 62; vs IUGR males, 1754 ± 70; sham females, 1802 ± 85; vs in IUGR females, 1736 ± 61, *p* > 0.05). A 2 h EdU pulse labeling of all dividing cells showed that IUGR offspring had a decreased percentage of proliferative cells in the VZ compared with shams in both sexes (*p* = 0.045; Fig. 2*B*). To assess proliferative cells that transition from the S to the M phase of the cell cycle within the 2 h EdU labeling, we colabeled them with pHH3 and EdU and found that IUGR offspring of both sexes had decreased mitosis at E15.5 (Figs. 2*C*). Surprisingly, IUGR offspring had an increased percentage of Tbr2^+^ INPs in the marginal zone (MZ), dentate migratory stream, and dentate fimbriodentate junction (FDJ) in the E15.5 DG compared with shams in both sexes (*p* = 0.038; Fig. 2*D*), suggesting premature neuronal differentiation of NSCs.

**Figure 3.**
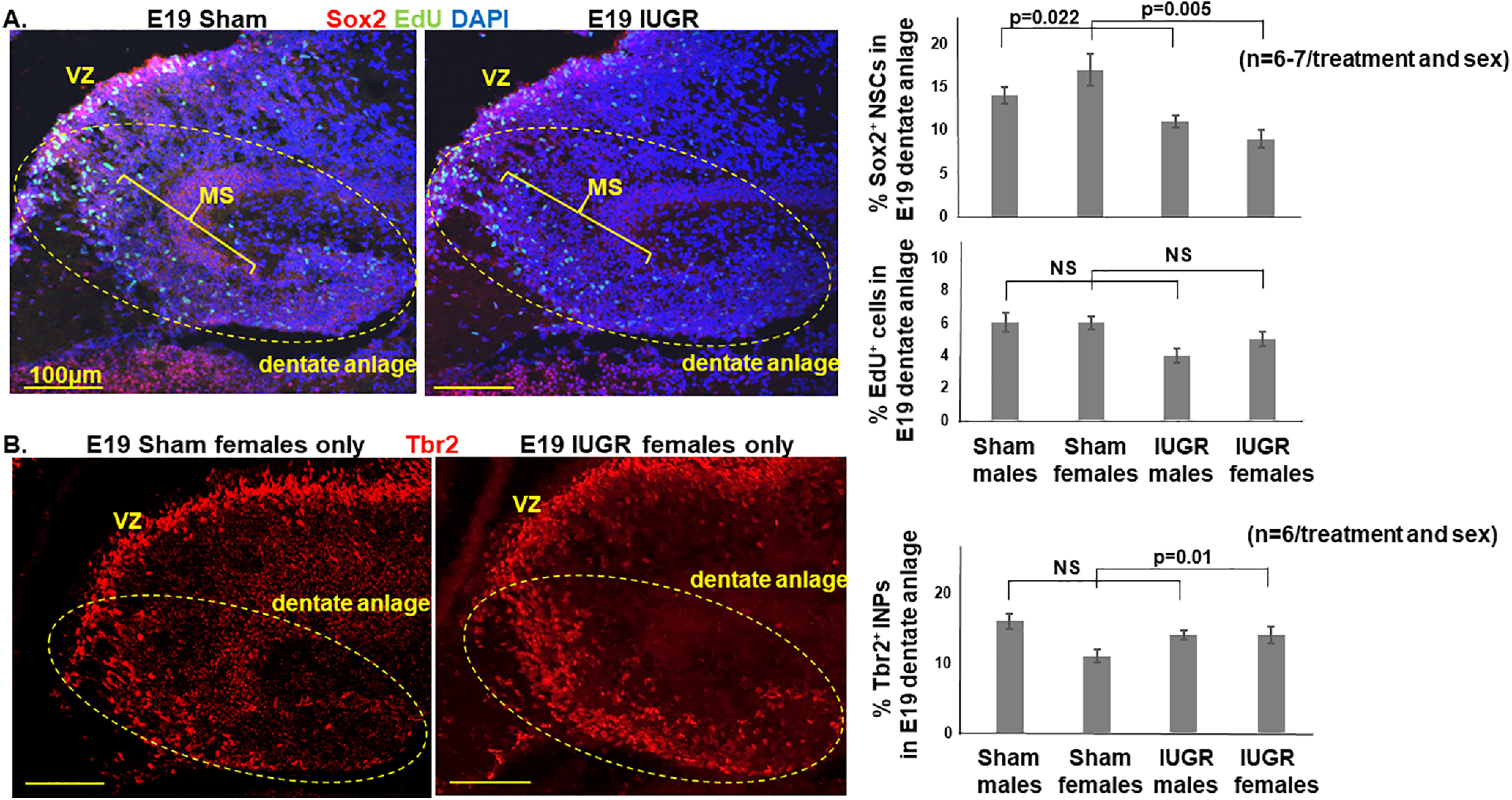
***A***, Representative photomicrographs of Sox2^+^ NSCs and EdU^+^ proliferative cells in E19 dentate anlage. Blue DAPI stain denoted cell nuclei. IUGR offspring of both sexes showed decreased percentages of Sox2^+^ NSCs in the VZ and migratory stream (MS). *p* = 0.022, IUGR males compared with sex-matched sham controls; *p* = 0.005, IUGR females compared with sex-matched sham controls. By contrast, IUGR offspring of both sexes showed similar percentages of EdU^+^ proliferative cells in VZ and migratory stream. Dotted eclipses delineate the area of dentate anlage manually traced in ImageJ. ***B***, Representative photomicrographs of Tbr2^+^ INPs in E19 dentate anlage. IUGR female offspring showed an increased percentage of Tbr2^+^ INPs in the MS and dentate anlage (*p* = 0.01, IUGR females compared with sham females). IUGR males had a percentage of Tbr2^+^ INPs similar to that of sham males. Two-way ANOVA with Tukey’s HSD *post hoc* test was used to examine the effects of treatment (sham or IUGR), sex, or both sex and treatment on the percentages of Sox2^+^, EdU^+^, or Tbr2^+^ cells.

**Figure 4.**
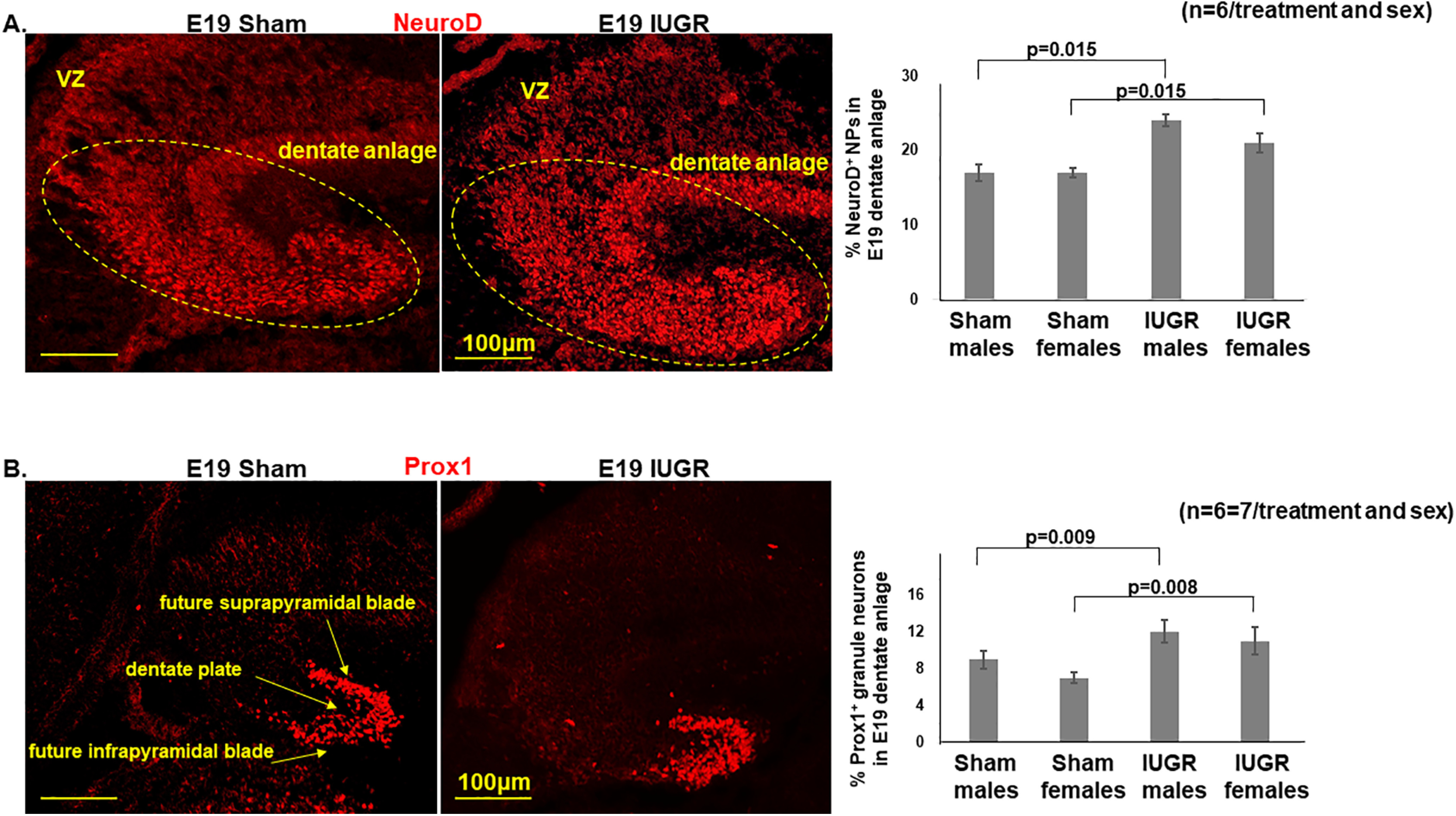
***A***, Representative photomicrographs of NeuroD^+^ NPs in E19 dentate anlage. IUGR offspring of both sexes had increased percentages of NeuroD^+^ NPs (number of NeuroD^+^ cells divided by number of DAPI^+^ cells) in dentate anlage (*p* = 0.015, IUGR males and females compared with sex-matched sham controls). ***B***, Representative photomicrographs of Prox1^+^ immature and mature glutamatergic granule neurons in E19 dentate anlage. IUGR offspring of both sexes had increased percentages of Prox1^+^ granule neurons (the number of Prox1^+^ cells divided by the number of DAPI^+^ cells) in dentate plate and granule cell layers of the future suprapyramidal and infrapyramidal blades (*p* = 0.009 and *p* = 0.008, respectively in IUGR males and females compared with sex-matched sham controls). Two-way ANOVA with Tukey’s HSD *post hoc* test was used to examine the effects of treatment (sham or IUGR), sex, or both sex and treatment on the percentages of NeuroD^+^ or Prox1^+^ cells.

By E19, IUGR offspring of both sexes continued to have decreased percentages of Sox2^+^ NSCs in the DG (*p* = 0.022; Fig. 3*A*), while proliferation was unaffected (Fig. 3*A*). Importantly, our quantification of proliferative cells included the ventricular zone, dentate migratory stream, and within the dentate anlage. IUGR females at this gestational age additionally showed increased percentages of Tbr2^+^ INPs (Fig. 3*B*), NeuroD^+^ NPs, and Prox1^+^ immature and mature granule neurons (Fig. 4*A*,*B*) compared with sham females. By contrast, IUGR males showed increased percentages of NeuroD^+^ NPs and Prox1^+^ granule neurons compared with sham males (Fig. 4*A*,*B*). In addition to increased percentages of neuronal progenitor cells and immature/mature neurons, the localization of these cells appeared more widely distributed across the dentate plate rather than confined to the future suprapyramidal or infrapyramidal blade or in the hilus (Fig. 4*B*).

### IUGR reduces postnatal hippocampal volumes P18 and P40 including DG volume at P40. IUGR reduces P18 DCX^+^ neuronal committed progenitor cell volumes but increases mean dendritic lengths

**Figure 5.**
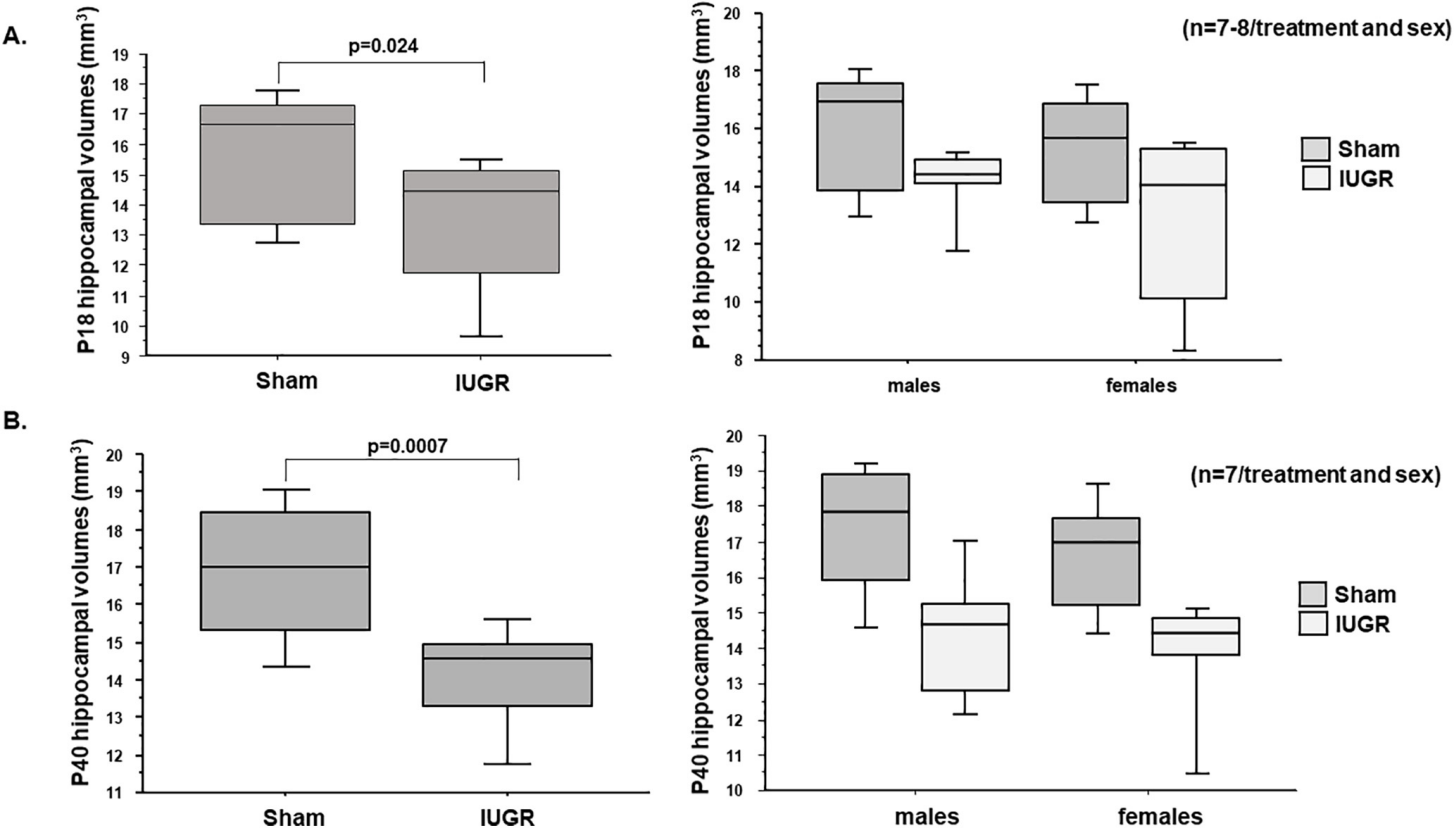
P18 and P40 hippocampal volumes (in mm^3^) including DG and CA regions. ***A***, IUGR decreased P18 hippocampal volumes in both males and females compared with sex-matched sham controls. *p* = 0.024, IUGR compared with sham controls. ***B***, IUGR also decreased P40 hippocampal volumes in both males and females compared with sex-matched shams. *p* = 0.0007, IUGR mice compared with sham controls. Sex differences in hippocampal volumes are shown to the right of ***A*** and ***B***. Two-way ANOVA with Tukey’s HSD *post hoc* test was used to examine the effects of treatment (sham or IUGR), sex, or both sex and treatment on hippocampal volumes.

Given that the murine hippocampal DG granule cell layer completes its development by approximately P30 (Seki et al., 2014); we investigated hippocampal volumes at P18 and P40 to track developmental progression of the entire structure. We found that IUGR diminished the overall hippocampal volumes at both ages (Fig. 5*A*,*B*).

**Figure 6.**
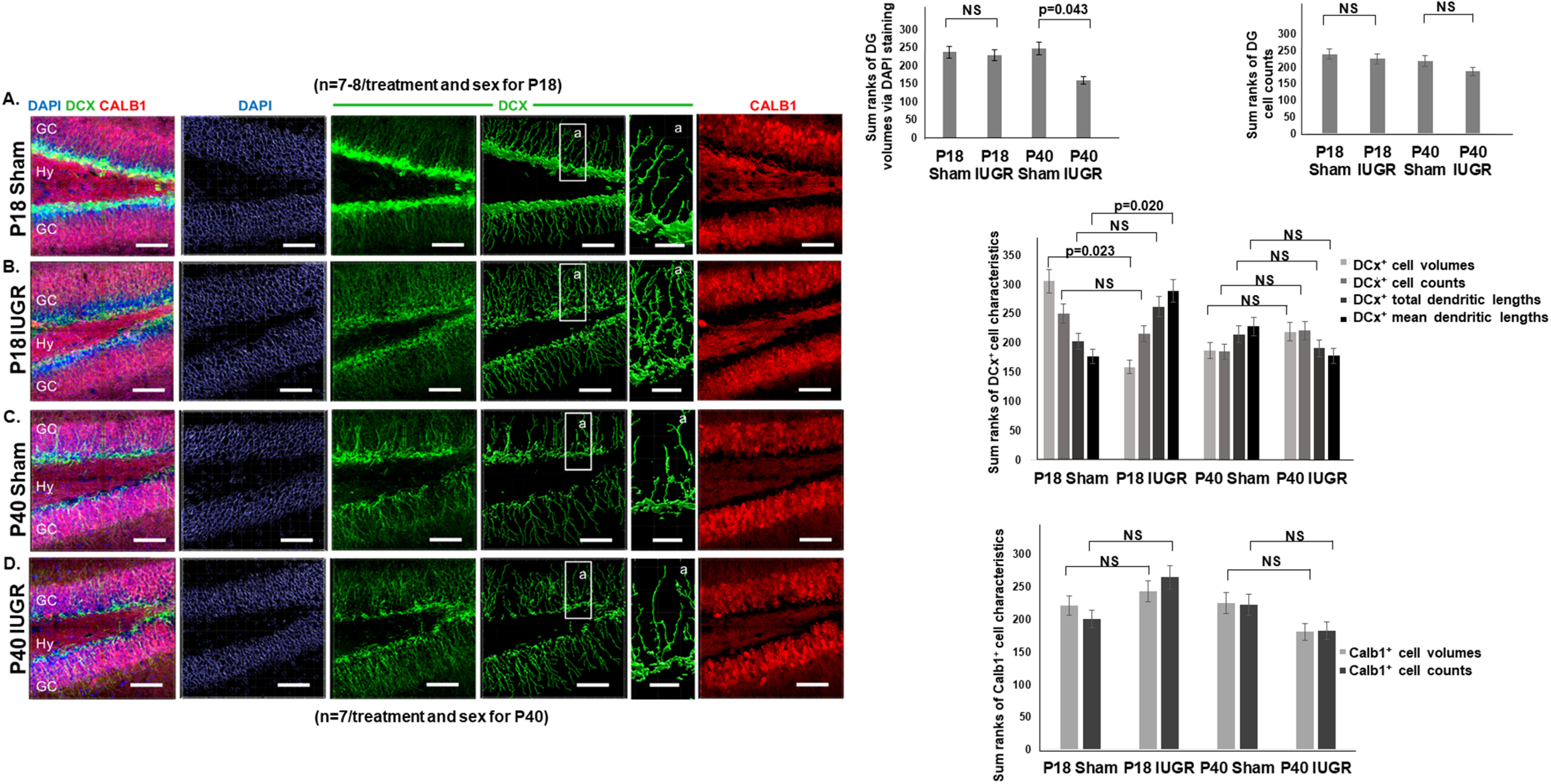
***A–D***, Representative photomicrographs of P18 (***A***, ***B***) and P40 (***C***, ***D***) hippocampal DG showing double-labeling of DCX and Calb1 immunofluorescent staining. DAPI was used as a counterstain for all cell nuclei that have been rendered non-nuclear under the Imaris “DAPI surface reconstruction” mode for quantification. GC, Granule cells; Hy, hilus. Within each DCX fluorescent image in ***A–D***, an enlarged region boxed as region a shows the DCX cell body volume and dendritic branching. Quantification of P18 sham and IUGR hippocampi using DAPI showed similar DG volumes. NS, No significance. Quantification of P40 sham and IUGR hippocampi however showed smaller DG volumes (*p* = 0.043). Quantification of DG cell counts showed no difference between sham and IUGR hippocampal DG at P18 or P40. Quantification of DCX^+^ cell characteristics showed that IUGR decreased P18 DCX^+^ cell volumes but increased mean dendritic lengths. Last, quantification of Calb1^+^ cell characteristics showed no differences in cell volumes or counts between sham and IUGR at P18 or P40 (NS). Given that these were all nonparametric variables, Mann–Whitney *U* test was used to compare sham and IUGR groups. No sex differences were noted.

We next quantified DCX^+^ neuronal committed progenitor cells/neuroblasts and Calb1^+^ neurons in P18 and P40 hippocampi. DCX is a microtubule binding protein that has high expression until mature neuronal markers take over (Brown et al., 2003). Calb1 expression, on the other hand, has been shown to affect hippocampal long-term potentiation and learning and to play a neuroprotective role in animal models of ischemic brain injury, and therefore subserves a critical role in adult function (Soontornniyomkij et al., 2012). We found that while the total number of DAPI^+^ cells was similar between sham and IUGR DG at P18 (Fig. 6*A*,*B*, and right panels), IUGR decreased the total number in P40 IUGR offspring in both sexes (Fig. 6*C*,*D*, and right panels). The total number of DG granule neurons within all DAPI^+^ cells was unchanged in either postnatal age (Fig. 6). Despite having no changes in DCX^+^ cell counts or total dendritic lengths at P18, IUGR led to decreased DCX^+^ cell volumes (Fig. 6*A*,*B*, and right panels) and increased mean dendritic lengths (Fig. 6*A*,*B*, and right panels). These changes normalized by P40 (Fig. 6*C*,*D*, and right panels). We found no changes in Calb1^+^ cell volumes or cell counts at P18 or P40 (Fig. 6).

### IUGR differentially expresses 611 protein-coding transcripts, of which the canonical and noncanonical Wnt signaling is predicted to have downregulated activity, whereas the glutamate receptor signaling is predicted to have upregulated activity

**Figure 7.**
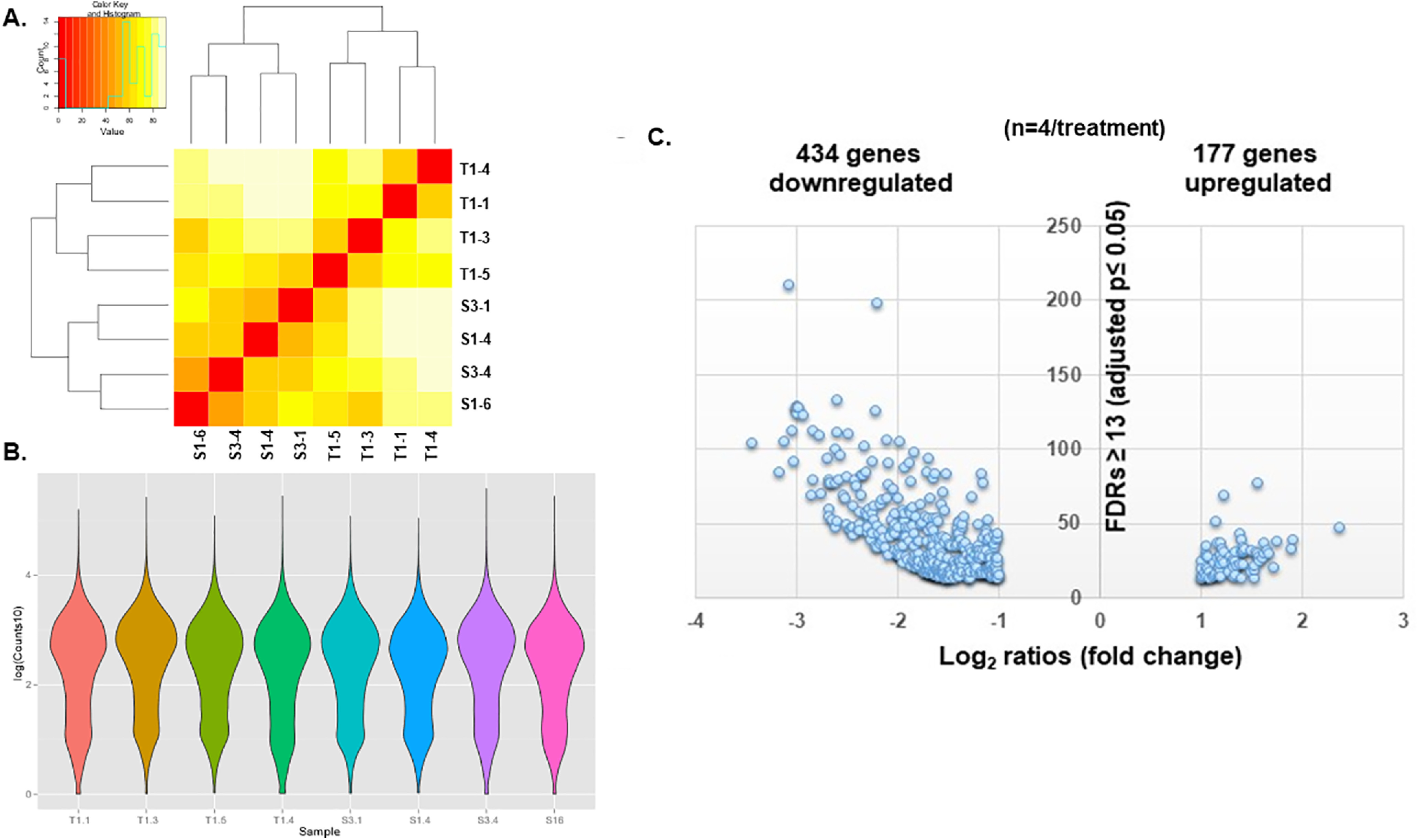
***A***, Heat map of sham and IUGR hippocampi used for RNA-seq showing the intersample similarities in gene expression within sham (S3-1, S1-4, S3-4, and S1-6) and IUGR (T1-4, T1-1, T1-3, T1-5) groups. Red-orange color signified sample similarities between two samples, whereas light yellow-white signified sample dissimilarities between two samples. ***B***, Violin plots of the counts in each sham or IUGR sample. The kernel density estimation shows that the distribution shapes between sham and IUGR data are similar. Wider sections of the violin plot represent a higher probability that certain genes will take on the given value, whereas the skinnier sections represent a lower probability. ***C***, Volcano plot of the 611 differentially expressed protein-coding gene transcripts in E15.5 IUGR hippocampus via RNA-seq. A total of 434 genes (∼70%) were downregulated. The *x*-axis denotes log_2_ ratios, which are fold changes in gene expression (log2 ratios greater than or equal to +1 or less than or equal to −1 = twofold increase or decrease). The *y*-axis denotes false discovery rates (FDRs) ≥ 13 = adjusted *p* ≤ 0.05.

Noting that IUGR promotes an imbalance toward neuronal differentiation over NSC self-renewal in embryonic life, we performed RNA sequencing of E15.5 sham control (S3-1, S1-4, S3-4, S1-6) and IUGR (T1-4, T1-1, T1-3, T1-5) hippocampi to determine which downstream genes may be responsible for the cellular phenotype (Fig. 7*A*,*B*). Of the 16,873 mouse genes analyzed, 611 protein-coding gene transcripts were differentially expressed (Fig. 7*C*). Approximately 70% of IUGR-affected genes were downregulated (434 genes). Ingenuity Pathway Analysis identified the canonical (Wnt/β-catenin) and noncanonical Wnt [planar cell polarity (PCP)] signaling pathways as predicted to have downregulated activity in E15.5 IUGR hippocampi (Fig. 8*A*, blue bars, Table 1). The glutamate receptor signaling pathway, which shared the same differentially expressed genes as the endocannabinoid neuronal synapse pathway, cAMP response element-binding protein (CREB) signaling, neuropathic pain signaling pathway, synaptic long-term potentiation, and synaptogenesis signaling pathway, was predicted to have upregulated activity (Fig. 8*A*, orange bars, Table 1).

**Table 1:** List of differentially expressed protein-coding gene transcripts between E15.5 sham and IUGR hippocampi

Gene pathways	Gene names	Fold changes
Wnt and PCP signaling	*Wnt2b*	↓3.8×
	*Wnt3a*	↓6×
	*Wnt8b*	↓2.7×
	*Wnt9a*	↓2.8×
	*Wnt10a*	↓3.3×
	*Dkk1*	↓5×
	*Wif1*	↓3.6×
	*Rspo1*, *2*, *3*	↓3.3×, 5.2×, 2.3×
	*Sp5*	↓2.1×
Endocannabinoid neuronal synapse/CREB signaling/glutamate receptor signaling/ neuropathic pain signaling/synaptic LTP/synaptogenesis signaling	*Grm1*, *5*, *8*	↑2.1×, 2.6×, 2.3×
	*Grin3a*	↑2.2×
	*Gng4*	↑3.4×

Wnt and PCP signaling pathways predicted to have downregulated activity included Wnt ligands (*Wnt2b*, *Wnt3a*, *Wnt8b*, *Wnt9a*, *Wnt10a*), Wnt inhibitors (*Dkk1* and *Wif1*), activators for Wnt and PCP pathways (*Rspo1*, *2*, *3*), and readout of Wnt pathway (*Sp5*). Corresponding fold changes in gene expression are shown. Glutamate receptor signaling plus other signaling pathways predicted to have upregulated activity included the metabotropic glutamate receptors (*Grm1*, *5*, *8*), glutamate ionotropic receptor NMDA type subunit 3a (*Grin3a*), and G-protein subunit γ 4 (*Gng4*).

Knowing the crucial role that Wnt signaling plays in maintaining stem and progenitor cell identity (Zhou et al., 2006), and, given the predicted downregulated activity of this pathway in IUGR, we validated the mRNA expression of these canonical and noncanonical Wnt pathway genes in a separate cohort of E15.5 sham control and IUGR hippocampi. Similar to our RNA-seq results, we found that *Wnt 2b* and *Wnt3a* gene expression was decreased (Fig. 8*B*). Similar decreases were detected in *R-Spondin 3* (*Rspo3*), which encodes a ligand for LGR4-6 receptors that activates the Wnt/PCP pathway; *Dkk1* and *Wif1*, which encode inhibitors of the Wnt pathway; and *Sp5*, which encodes a transcription factor and is often used as a readout of the Wnt pathway (Wodarz and Nusse, 1998). *Fdz7*, which encodes a receptor that binds Wnt ligands, in contrast, was unaltered in both RNA-seq and qRT-PCR.

## Discussion

Children born with IUGR are at increased risk for cognitive impairment among many other negative health outcomes (Barker, 2004; van Wassenaer, 2005; Chen et al., 2016). One specific affected area of cognitive impairment is in learning and memory, which is mediated in part by the hippocampus. Despite this known risk, no human or animal studies exist to describe the *in utero* hippocampal changes that lead to postnatal learning and memory deficits. Sexual differences in hippocampal neurogenesis are also not well described. Such a lack of knowledge not only impedes our ability to counsel families about mechanisms of disease, but also hinders our ability to design therapy targeted at improving learning and memory function in these at-risk children. Recognizing this gap, our laboratory developed a mouse model of IUGR that closely mimics many features of human IUGR (Fung et al., 2011, 2015, 2016; McKnight et al., 2015; Gibbins et al., 2018) to dissect the functional, cellular, and molecular phenotypes of the developing IUGR hippocampus.

Behavioral testing of our 3- to 6-month-old IUGR mice revealed marked memory deficits represented by a lack of object recognition, contextual memory, and cued memory during short-term recall. This echoes what is known in children born with IUGR who have been described to have poor memory in childhood and adulthood in multiple studies (Hadders-Algra and Touwen, 1990; Geva et al., 2006; Leitner et al., 2007; Guellec et al., 2011). We find it intriguing that IUGR females from this hypertensive disease of pregnancy model appeared to perform poorer than IUGR males. The reasons behind this sexual dimorphism are at present unclear. However, we posit that a delay in catch-up growth in IUGR females versus IUGR males (77 vs 28 d) may be partly responsible. P15 to P30 is a critical period of hippocampal dendritogenesis and synaptogenesis; thus, delayed catch-up growth in IUGR females likely missed this critical window of development, which could account for the sex differences. We also saw the smallest hippocampal volumes in IUGR females, which may also contribute to further compromise. Many studies in humans and experimental models with IUGR have shown that poorer catch-up growth is associated with neurodevelopmental deficits, whereas faster catch-up growth is neuroprotective but predisposes offspring to early-onset metabolic syndrome (Yeung, 2006; Jimenez-Chillaron and Patti, 2007). It is also well established that a positive correlation exists where a larger reduction in hippocampal volume signifies worse memory performance (Isaacs et al., 2000; Mallard et al., 2000; Lodygensky et al., 2008; Camprubí Camprubí et al., 2017). We additionally postulate that IUGR male and female offspring may have a different brain sex steroid hormone balance, particularly with regard to progesterone and its neuroprotective metabolite, allopregnanolone, where IUGR males in a guinea pig model have been shown to have higher brain levels of 5α-reductase, which promotes allopregnanolone synthesis (Kelleher et al., 2011).

With the demonstration of learning and memory deficits in our model, we next pursued a characterization of the effects of IUGR on embryonic hippocampal DG neurogenesis as well as postnatal neuronal development to understand the *in utero* events that set the stage for lifelong learning and memory impairment. We focused on the DG because its granule neurons receive primary afferent input from the entorhinal cortex for memory formation and are the first relay station projecting information to the CA regions. DG and CA3 connectivity forms the Mossy Fiber pathway, which is responsible for learning and memory. DG granule neurons also arise from a distinct NSPC pool from CA pyramidal neurons, with their development regulated by intrinsic factors and extracellular signaling pathways that drive proliferation and differentiation of embryonic neural precursors (Urbán and Guillemot, 2014).

Correlating the timing of maternal hypertension in our model to the timing of embryonic DG neurogenesis in mice, we found that IUGR decreased the percentage of Sox2^+^ NSCs in the ventricular zone of the cortical hem along with decreased proliferation. Contrary to our expectation, IUGR increased the percentage of Tbr2^+^ INPs that populated the marginal zone and migratory stream toward the fimbriodentate junction. Collectively, these findings suggest that Sox2^+^ NSCs are likely precociously exiting cell cycle and becoming committed to Tbr2^+^ INPs as a result of IUGR. This is supported by decreased phosphohistone H3 staining or mitosis amid our overall decreased EdU^+^ proliferative stem/progenitor cells in the IUGR DG. In other words, the IUGR milieu may be directing Sox2^+^ NSCs to exit the cell cycle and becoming committed to a neuronal lineage rather than continuing in a proliferative state. As maternal hypertension continued into late gestation, the percentage of Sox2^+^ NSCs in the IUGR DG remained diminished through birth. The fewer Sox2^+^ NSCs, however, retained a proliferative capacity similar to that of sham NSCs at this age. It remains to be determined what the long-term consequences are with such embryonic NSC depletion, but, given that adult DG neurogenesis is dependent on the endowment of the embryonic stem cell pool (Urbán and Guillemot, 2014) plus that learning can induce neurogenesis in adult animals, postnatal effects on that process are possible. In addition, we found that IUGR males showed a slightly faster maturation of the neuronal lineage into NeuroD^+^ NPs and Prox1^+^ granule cells, whereas IUGR females displayed the entire spectrum of neurogenic markers. Our finding of accelerated neurogenesis was indeed surprising given that postnatal studies in other IUGR animal models have shown decreased neuron number with altered dendritic–axonal connections (Mallard et al., 2000; Lister et al., 2005; Fung et al., 2012). Whether these prematurely generated neurons survive and integrate properly into circuitry or instead undergo apoptosis are being investigated.

These embryonic findings in the hippocampal DG are novel. While IUGR has been experimentally induced in rats, guinea pigs, rabbits, and sheep for many years (Nitsos and Rees, 1990; Mallard et al., 1998, 2000; Tashima et al., 2001; Dieni and Rees, 2003; Eixarch et al., 2012; Fung et al., 2012; Illa et al., 2013; Miller et al., 2014; Basilious et al., 2015), longitudinal examination of embryonic brain development is clearly under-researched in the field. Other brain regions known to be susceptible to IUGR injury include the cerebral cortex. In a rat model of IUGR induced by bilateral uterine artery ligation at E17 through E22, morphologic brain changes via Nissl staining at different timepoints during prenatal and postnatal periods showed significant cell loss and disturbance in the proliferation and migration of existing neurons that was first evident antenatally and persisted for at least 10 weeks postnatally in IUGR rats (Tashima et al., 2001). Open field test additionally revealed locomotor disturbance at P49 in IUGR male rats but not in IUGR female rats or control rats (Tashima et al., 2001). In another rat model of IUGR induced via protein calorie restriction from E1 through delivery, transmission electron microscopy reported sparse brain cortex structures in newborn rats showing many scattered apoptotic cells, a decreased number of synapses, lower glial cell proliferation, and fewer neurons that were sparsely arranged compared with control rats (Liu et al., 2011). Deficits in neuronal connectivity were also apparent, with term equivalent IUGR fetal sheep demonstrating a 17% decrease in synaptic density within the cerebral cortex (Bisignano and Rees, 1988). In addition, cell proliferation zones showed decreased expression of antiapoptotic protein Bcl-2, while proapoptotic p53 immunoreactivity was increased (Uysal et al., 2008).

The work in our current postnatal studies would suggest that NSCs that endow the postnatal DG develop in an altered trajectory after IUGR. In the mammalian brain, activated NSCs in the DG give rise to proliferating neural progenitor cells, differentiate to DCX^+^ neuroblasts, which gradually mature to terminally differentiated neurons (Knotek et al., 2020). During mid-development of postnatal DG, DCX^+^ neuroblasts have smaller cell volumes with higher mean dendritic lengths in our IUGR offspring. This suggests that the prenatally endowed DG NSCs continue to show a degree of advanced maturation, which will likely impact new dendritic–axonal connections and alter learning and memory function. Although the DCX^+^ cell changes detected in P18 are abrogated after the completion of DG development, our IUGR offspring are clearly affected in their short-term learning ability again supported by the overall decreased hippocampal volumes at P18 and P40.

In search of the molecular pathways responsible for our observed phenotypes of accelerated neurogenesis and embryonic NSC depletion, we have identified the canonical and noncanonical Wnt signaling pathways as logical candidates. Both pathways require Wnt ligand binding to cognate receptor types such as the Frizzled proteins, but the two downstream pathways diverge substantially. Our RNA-seq data identified several *Wnt* genes with decreased expression in IUGR. Of these, *Wnt3a* is of notable significance not only because of its greatest decrease in expression, but also because beginning at E9.75 in mice, it marks the cortical hem from which induction and patterning of the hippocampus originates (Roelink and Nusse, 1991; Grove et al., 1998). In *Wnt-3a* mutants, medial hippocampal fields are absent and lateral hippocampal fields are severely reduced because of the lack of proliferative expansion of caudomedial stem and progenitor cells. In the case of IUGR, *Wnt3a* reduction could lead to reduction of fewer Sox2^+^ NSCs because of decreased progenitor proliferation. Further supporting the relevance of Wnt signaling in IUGR, we also found decreased expression of *Dkk1* and *Wif1*, which encode inhibitors of the Wnt pathway. These genes could be downregulated as a result of reduced feedback from the overall decrease in Wnt signaling that is evident from decreased *Sp5* expression, which is possibly a way to allow remaining Wnt ligands to maintain residual Wnt pathway activity.

Consistent with our observation of decreased NSC proliferation, premature cell cycle exit could drive progenitors in the IUGR brain to differentiate toward the neuronal lineage, indicated by a significant increase in Tbr2^+^ INPs. Previous work demonstrated that Tbr2 is critically required for DG neurogenesis in both developing and adult mice (Hodge et al., 2012). In the absence of Tbr2, INPs are depleted despite augmented NSC proliferation. Of further importance, this study also found that Tbr2 is enriched at T-box binding sites in the *Sox2* locus to repress *Sox2* expression, suggesting that Tbr2 may promote progression from multipotent NSCs to neuronal-specified INPs through this mechanism. Once the transition to Tbr2 is established, neuronal maturation appears to proceed in an unhindered fashion in IUGR.

The increase in expression of glutamatergic receptor signaling components that we identified in our RNA-seq data could partially explain the accelerated neurogenesis phenotype in our model, but would not likely account for the NSC depletion phenotype. Such an increase in signaling could also simply represent an overabundance of developing glutamatergic DG granule neurons. Alternatively, an actual signaling increase within cells could provide a mechanism for the immature DG granule neurons to survive in IUGR. In the developing brain, classical neurotransmitters such as glutamate exert trophic effects before synaptogenesis (Ye et al., 2005; Karten et al., 2006), and the immature noncontacted cells might express functional glutamate receptors for this purpose. Our RNA-seq data showing increased glutamate receptor signaling could be consistent with DG granule neuron maturation and survival after NSC cell cycle exit.

Neurons are also believed to be the cells most vulnerable to an ischemic insult. In the setting of IUGR where uteroplacental insufficiency occurs, ischemic insult may actually represent multiple insults to include hypoxic, oxidative, and glutamate stresses (Kitagawa, 2007). Glutamate stress is unique to the brain in that ischemia induces release of glutamate into the extracellular space, which in turn binds to ionotropic glutamate receptors such as NMDA and AMPA receptors. Activation of glutamate receptors causes a calcium influx that activates Ca2^+^/CaM kinase, the phosphoinositide 3-kinase, and Ras/MAPK cascades, which then phosphorylate several transcription factors including CREB. Activation of CREB leads to the expression of survival genes such as Bcl-2 and brain-derived neurotrophic factor (BDNF). BDNF is critical to modulate synaptic plasticity, specifically long-term potentiation and long-term depression, which is thought to be the underlying cellular mechanism for learning and memory processes (Schmidt et al., 2013). Although not yet investigated in our model, increased BDNF signaling in embryonic life could at least provide a survival mechanism for the prematurely differentiated neuronal progenitors.

Clearly, much work must be done to define the roles of glutamatergic signaling in the IUGR brain, particularly as it relates to embryonic development in a perinatal insult. The complexity of both ionotropic and metabotropic glutamate receptor signaling is much better delineated in the postnatal brain where presynaptic and postsynaptic connections are complete. Even then, the overlapping expression patterns of these glutamate receptors vary between cells, but also between individual synapses, and their activity is context dependent (Reiner and Levitz, 2018).

In summary, our behavioral, cellular, and molecular data lead us to conclude that short-term memory deficits in adult IUGR offspring may be associated with aberrant hippocampal DG neurogenesis via decreased Wnt signaling in prenatal life. Downregulation of Wnt signaling in the face of uteroplacental insufficiency could promote cell cycle exit and premature commitment to neuronal differentiation. Doublecortin^+^ neuronal progenitors/neuroblasts in the postnatal DG continue to display accelerated maturation via increased dendritic branching, occurring at a time when dendritic–axonal connections are being established. The ultimate outcome of these developmental aberrations would explain the effect of reduced hippocampal volume and impaired learning and memory function on short-term recall in adulthood. While accelerated differentiation may be an adaptive response of the fetus to ensure survival by producing more neurons, because DG granule neuron generation continues in the first postnatal month in mice, precocious neurogenesis could result in neuronal apoptosis, improper neuronal integration into circuitry, as well as continued exhaustion of NSCs, which are needed for an expanding tissue such as the growing hippocampus. If we are able to validate decreased Wnt signaling as the underlying basis of IUGR-induced aberrant DG neurogenesis, future therapeutic intervention aimed at augmenting Wnt signaling may provide an avenue to negate premature neuronal differentiation and NSC depletion in IUGR offspring and to ameliorate learning and memory deficits.

## Synthesis

Reviewing Editor: Lynda Erskine, University of Aberdeen Decisions are customarily a result of the Reviewing Editor and the peer reviewers coming together and discussing their recommendations until a consensus is reached. When revisions are invited, a fact-based synthesis statement explaining their decision and outlining what is needed to prepare a revision will be listed below. The following reviewer(s) agreed to reveal their identity: Phu Tran. Note: If this manuscript was transferred from JNeurosci and a decision was made to accept the manuscript without peer review, a brief statement to this effect will instead be what is listed below.

Your manuscript has been reviewed by two experts in the field who are agreed that the findings are interesting and valuable to the community. The data provide evidence that IUGR impairs neurogenesis during early stages of hippocampal development with changes in neural stem cell populations. In addition, the paper provides potential mechanisms by which these cellular changes can act to produce deficits in learning and memory in later life. Whilst these findings are important and interesting, we are all agreed that additional experiments are required to strengthen the manuscript, as well as more in-depth analyses and discussions in the context of other IUGR models.

Below is a synthesis of the reviewers’ comments that, following discussion, we are all agree need to be addressed before the manuscript can be accepted for publication.

Major points:

1. A major problem in this manuscript is that the authors do not examine if neurogenesis is impaired in the adult DG. They perform behavioural tests in mice that are 3-6 months old and could very easily analyse the number of NSCs and INPs in the DG of these mice.

Is neurogenesis impaired in adult IUGR mice? Does IUGR lead to a smaller DG?

2. The authors should provide the BW of the adult IUGR mice to illustrate whether there was significant postnatal catch-up growth, which could contribute to changes affecting hippocampal plasticity.

3. What area of the developing DG was analysed for cell counting? It is unclear from reading text or looking at the images which area was quantified. How did the authors decide which part of the ventricular zone to quantify? Did the authors only analyse the dentate neuroepithelium or the ventricular zone in hippocampal neuroepithelium?

It would be helpful if the made an illustration to clarify the areas of the developing dentate they counted. Additionally, the authors say that they analysed the “dorsal DG”. How do they identify the dorsal dg at E15.5 and E18.5?

I think it’s a bit misleading calling it the DG at E15.5 and E18.5 when there is no discernible dentate structure, dentate primordium or anlage would be more accurate.

4. While the authors indicated that 2-way ANOVA was employed, no description of the main effect was provided in the text. It was not clear whether posthoc statistical tests were corrected for multiple comparisons. Additionally, the authors should be consistent with the way they present statistical significance throughout the paper, either show the P-value on the graph or have a star system where each star indicates a range of values. The authors also should indicate in the figure legends which statistical test was done.

5. Ingenuity Pathway Analysis. A detail description of this analysis is needed, including Z-core selection.

6. An in-depth discussion is needed, especially in the context of other IUGR models. While the focus of the study was on the developing hippocampus, a discussion of possible and similar changes in other brain structures projecting to and from the hippocampus is warranted. An extended discussion of the gain of activity in signaling pathways (Glutamate receptor/CREB/LTD) in the context of increased number of differentiated cells as well as learning impairments is needed.

Comments on the Figures:

1. Combine Fig. 1 and 2; change asterisks that denote significant differences between groups

2. Fig.3 - A higher magnification image is needed for Sox2+ counterstained with DAPI given this was a major finding of the paper. Were there sex differences in these findings?

3. Fig.4 - Provide some landmarks in the fig. panels. Increase gain for Sox+ cells, which are hard to see in the current image. Reorganize graphs for better clarity. Panels b-d: are these from female mouse embryos? They were described as both sexes in text.

Lines 263-275: Fig.4a left and right showed sham and IUGR images, not difference between Sox2+ cells and proliferative cells. In addition, both sexes showed gain in Tbr and NeuroD; how are they in contrast as described in text?

Is the difference between sham male and sham female in the number of Tbr2+ cells significant?

4. Fig.5: Provide a Wnt signaling pathway with specific gene changes along the pathway. Panel b - were these pathways selected by Z-score {greater than or equal to} 2? A description for gain of functions (e.g., glutamate receptor signaling) is needed.

Other points:

1. Were images blinded for quantification?

2. Were the brains cut coronally? Please specify in methods

3. Some parts of the text are not referenced correctly. They cite Seki et al 2014, to support the statement “it has a distinct NSPC pool known to be regulated by environmental signals throughout life”. This paper examines the development of the DG, and do not look at environmental signals.

Altman and Bayer, 1990b, a; Saxe et al., 2006; Jessberger et al., 2009 are cited to support that “DG neurogenesis in the perinatal and adult periods is strongly associated with learning and memory deficits”. Saxe and Jessberger only deal with adult neurogenesis, and the Altman papers do not deal with learning and memory. The authors need to add reference that “perinatal DG neurogenesis is associated with learning and memory”.

4. The authors need to indicate that they will deposit the RNAseq data into the NCBI database.

5. It would add import to the paper if double labeling (EdU + phosphorylated histone H3) data could be collected and present. The finding would reveal the proportion of actively prolifating cells in control vs IUGR embryos. Although the authors cited previous studies showing reduced cell number in adult mice that were IUGR; it would be informative to show such an effect in this IUGR model, demonstrating the generality of the finding.
